# The Liabilities of Hospital Trustees

**Published:** 1898-12-31

**Authors:** 


### THE LIABILITIES OF HOSPITAL TRUSTEES.

By a Barrister.

We are indebted to a contemporary for a note of a recent decision of the American Courts, which raises a question of the highest interest and importance to the trustees and managers of hospitals. Although not based upon any English case exactly in point, it was decided in accordance with the principles of our law as expounded in the House of Lords, and therefore it forms a precedent which would undoubtedly receive all possible respect if cited before our tribunals. It is not a little remarkable that the question involved has not hitherto been litigated in this country. At any rate, there is no reported case; though we believe the circumstances formed the subject of an action some few years ago, which was determined upon the particular facts, and therefore is no authority to which reference can be made. The point of the American case was very shortly this. Can a patient who is treated gratuitously at a hospital maintain any action against the governing body of the hospital for negligent treatment by any of the physicians or surgeons employed by the hospital? The reply which would suggest itself to many persons is in the negative. How, it may be asked, can a person who is treated gratuitously have any right to sue those who have provided him gratis with professional attendance for the negligence of those who have attended him. Such an argument, however, will not, we fear, hold water. It is a fallacy to suppose that, because a person renders service to another gratuitously, he thereby escapes all liability for the consequences of his acts. This is most strongly illustrated in the case of professional services such as are rendered by a physician or surgeon. The doctor, by undertaking to treat a patient, induces in him a confidence in his own skill which is a sufficient legal consideration to create a duty in the performance of the service rendered, and he is therefore liable to the patient for negligence. Now, the hospital authorities stand in the same legal position. They undertake to provide medical and surgical attendance for patients, and as, being a species of corporation, they cannot act personally, they act by their officers, of whom the hospital doctor must be deemed one. They may act gratuitously in the sense that they are neither paid by the patient nor by anyone else for the accommodation and services they provide, but they are, as was explained by the American judges, in the same position as a charitably-disposed individual, and can claim no greater exemption from liability. The hospital authorities hold out to the public, in effect, that they will provide proper medical and surgical advice, and thereby create a confidence in them, which imposes a corresponding obligation. The liability of the governors is, of course, a corporate one. The general funds of the hospital are answerable for the payment of compensation to the injured patient, who may at the same time have his remedy against the doctor who actually attended him. Such is the rule to be deduced from the case referred to, which is entirely in harmony with the principles of our law. It will no doubt be a serious matter for the governing bodies of hospitals, hampered as they already are for want of funds, if they are to be liable to be shot at by ex-patients generally; but it is as well to know the worst, and the knowledge of the responsibility which is incurred towards a patient, although a non-paying one, should tend to keep up the efficiency of our hospitals, which might suffer from the conviction that they enjoyed a special exemption on public grounds. It was said that if hospitals are to be liable in these cases the public would withhold subscriptions for fear that they might be diverted from the objects intended by the subscribers, which was not to provide a damage fund for speculative litigants, but to maintain a sufficient staff and equipment. This, however, is obviously an argument to be addressed to the Legislature rather than to the Courts. We have put the case against the hospital in perhaps a somewhat naked light; but we do not believe in maintaining the law to be what we wish rather than what it is. If it is defective, let us endeavour to get it amended. Meanwhile, forewarned is forearmed. The best safeguard, after all, is the high ideal which our hospitals have always endeavoured to attain to, and in this they need not fear comparison with any public or charitable institution. We may add that in the American case a further question of considerable professional interest was involved, viz., whether a child born deformed through alleged improper treatment before birth at a maternity hospital would have any right of action against the hospital? The problem thus presented is too difficult to be dealt with in the limits of the present article, but we hope to discuss it on another occasion.

